# Immune Responses to Anti-Hepatitis C Virus Antibodies during Pre-Liver Transplantation Direct-Acting Antiviral Therapy in Hepatitis C Virus-Infected Recipients Associated with Post-Liver Transplantation Allograft Injury

**DOI:** 10.3390/antib13010007

**Published:** 2024-01-16

**Authors:** Shu-Hsien Lin, Kun-Ta Wu, Chih-Chi Wang, Kuang-Tzu Huang, Li-Wen Hsu, Hock-Liew Eng, King-Wah Chiu

**Affiliations:** 1 Division of Hepato-Gastroenterology, Department of Internal Medicine, Kaohsiung Chang Gung Memorial Hospital, Kaohsiung 83301, Taiwan; susan77628@cgmh.org.tw; 2 Liver Transplantation Center, Department of Surgery, Kaohsiung Chang Gung Memorial Hospital, Kaohsiung 83301, Taiwan; ufel4996@ms26.hinet.net (C.-C.W.); huangkt@cgmh.org.tw (K.-T.H.); hsuliwen@ms55.hinet.net (L.-W.H.); eng6166.hsing@msa.hinet.net (H.-L.E.); 3 E-Da Healthcare Group, E-Da Hospital, College of Medicine, I-Shou University, Kaohsiung 82445, Taiwan; dixon034inmc@gmail.com; 4 College of Medicine, Chang Gung University, Taoyuan 33302, Taiwan; 5 Division of General Surgery, Department of Surgery, Kaohsiung Chang Gung Memorial Hospital, Kaohsiung 83301, Taiwan; 6 Institute for Translational Research in Biomedicine, Kaohsiung Chang Gung Memorial Hospital, Kaohsiung 83301, Taiwan; 7 Department of Pathology, Kaohsiung Chang Gung Memorial Hospital, Kaohsiung 83301, Taiwan

**Keywords:** acute cellular rejection, anti-HCV antibody, biliary complications, direct-acting antivirals, hepatitis C virus, liver transplantation

## Abstract

Background and Aims: The impact of antibody responses following direct-acting antiviral (DAA) therapy in hepatitis C virus (HCV)-infected recipients before and after liver transplantation (LT) is still undetermined. Methods: In this observational cohort study, we aimed to explore the association between changes in anti-HCV antibody titers following pre-LT DAA therapy and allograft injury, including biliary complications (BCs) and acute cellular rejection (ACR). Results: A total of 153 cases were enrolled from January 2015 to February 2021. Serum anti-HCV antibody titers were assessed before and after (day 30) LT. Among all recipients, 31/153 (20.3%) had pre-LT DAA therapy (the DAA group) and 122/153 (79.7%) did not undergo pre-LT DAA therapy (the DAA-naïve group). A higher incidence of post-LT BCs was observed in the DAA group (*p* = 0.028). Compared with the DAA-naïve group, the DAA group had a significantly higher mean level of anti-HCV titer upregulation (*p* = 0.0024); furthermore, among the recipients with BCs (n = 28) and ACR (n = 41), those in the DAA group exhibited significantly higher mean levels of anti-HCV antibody titer upregulation (*p* < 0.005). Conclusions: In conclusion, we speculate that the upregulation of anti-HCV antibody titers, which might have been induced via the restoration of HCV-specific immune responses through pre-LT DAA therapy, was associated with post-LT allograft injury.

## 1. Introduction

Hepatitis C virus (HCV) infection has decreased dramatically in Western countries, but end-stage liver disease associated with previous liver damage is one of the leading indications for liver transplantation (LT) worldwide [1]. Because of the availability of highly effective anti-HCV agents with direct-acting antivirals (DAAs), the composition of the LT waiting list has changed considerably; DAA use has also resulted in an improvement in post-LT outcomes, including lower likelihoods of graft failure, death, and retransplant procedures [2,3].

In formal pre-LT evaluation protocols, a series of laboratory tests, particularly viral serological tests, such as those for hepatitis A, B, and C, are performed to confirm the irreversible nature of a patient’s liver disease [4,5]. Anti-HCV antibody titers are utilized as a serological screening marker in the clinical diagnosis of HCV infection. A positive serum anti-HCV antibody test and negative polymerase chain reaction (PCR) test for HCV RNA indicate no evidence of current (active) HCV infection [6]; however, current serological tests for anti-HCV antibodies only detect antibodies to HCV antigens, which are unrelated to protection from HCV [7]. Moreover, recent clinical research has shown that viral clearance via DAA treatment does not eliminate HCV-related extrahepatic diseases, such as B-cell lymphoma and mixed cryoglobulinemia (MC), which result from the dysregulation of immune functions, particularly B-cells, abnormal lymphoproliferation, and the production of autoantibodies [8]. The causes and molecular mechanisms of these antibody responses to HCV infection have remained poorly understood.

The role of anti-HCV antibodies and their association with the dysregulation of immune functions have been investigated. Toyoda et al. (2005) reported that anti-HCV antibody titers decreased during a 10-year follow-up following the eradication of HCV through interferon therapy [9]. The mechanisms underlying the alteration in serum anti-HCV antibody levels have yet to be clarified. A previous study conducted on a Taiwanese population concluded that anti-HCV titers strongly predicted HCV viremia; this excellent performance could be generalized to either HCV mono-infected or HBV dually infected patients [10]. The association between changes in anti-HCV antibody titers and immune status in patients with sustained virologic response requires further investigation, especially in the era of DAAs. Although recent breakthroughs in research on antibody responses to chronic HCV infection have provided valuable insights into neutralizing antibodies, several crucial aspects of such responses must still be uncovered [11,12]. HCV-specific T-cell responses and humoral immunity have been reported to be associated with controlling HCV infection [13,14]. Moreover, T-cell dysfunction and exhaustion induced via immune tolerance have been demonstrated to be strongly associated with successful LT outcomes [15,16]. However, most studies have demonstrated that DAA treatment for HCV infection may result in, at least, the partial restoration of T-cell immune function [17]. Few studies, nevertheless, have examined whether pre-LT DAA therapy contributes to the restoration of immune function and, thus, affects post-LT graft injury.

In liver transplantation settings, biliary complications (BCs) and acute cellular rejection (ACR) often lead to post-transplant acute jaundice or graft dysfunction. In our previous study, we demonstrated that liver graft microRNA expression associated with different etiologies of post-LT acute jaundice includes acute rejection and cholangitis, recurrent hepatitis, and non-specific pathological and fatty changes [18]. However, we have not investigated the immune microenvironment of damaged liver grafts, especially in HCV-infected recipients.

It is unclear whether fluctuations in anti-HCV antibody titers before and after LT reflect the altered immune response of HCV-infected patients. Thus, the aim of this study was to explore the association between changes in anti-HCV antibody titers in chronic HCV patients who received pre-LT DAA therapy and allograft injury, including BCs and ACR, following LT.

## 2. Materials and Methods

### 2.1. Study Population and Study Design

**Figure 1:**
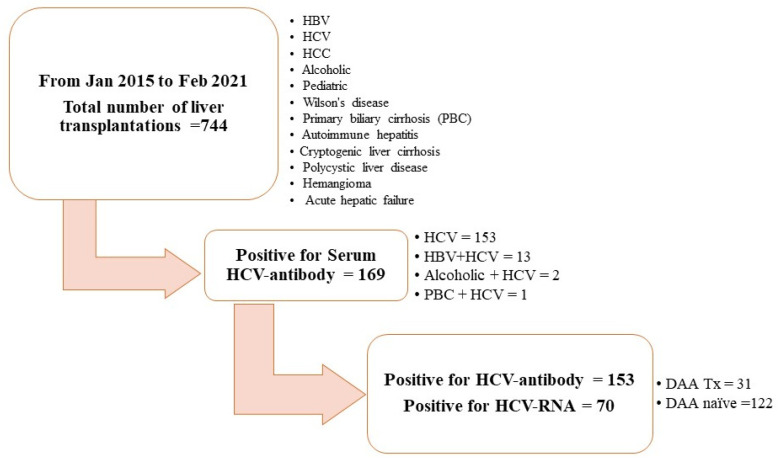
Flow chart of patient selection and exclusion.

In this observational cohort study, we selected 153 liver allograft recipients with positive serum anti-HCV antibody titers detected during pre-LT evaluation from January 2015 to February 2021 from our liver transplantation program. During this period, a total of 744 cases met our criteria for further screening. Among 169 recipients with positive serum results for anti-HCV antibodies, 13 cases with positive serum HBsAg, 2 cases with alcoholic history, and 1 with primary biliary cirrhosis were excluded from our study. Furthermore, we also excluded those with negative serum anti-HCV antibodies, positive serum HBsAg levels, underlying psychiatric conditions, primary biliary cirrhosis, or alcohol-related liver disease, in addition to those with a pediatric LT history (see Figure 1).

### 2.2. Pre-LT DAA Therapy

Among 153 recipients, 31 (20.3%) were administered pre-transplant DAAs, including Harvoni (i.e., 400 mg of sofosbuvir plus 90 mg of ledipasvir), or a combination of 400 mg of sofosbuvir/60 mg of daclatasvir/800 mg of ribavirin (defined as the DAA group), for 3 months based on their HCV genotype; the other 122 (79.7%) recipients did not have pre-transplant DAAs (defined as the DAA-naïve group) [19]. The duration between patients receiving DAA therapy and LT was approximately 3 to 6 months. All transplant recipients were followed up until September 2021.

### 2.3. Sample

Serial blood samples collected from all HCV-infected recipients with 3 mL of whole blood were tested for anti-HCV antibodies (using electrochemiluminescence immunoassays (ECLIAs)) and serum extraction for HCV RNA viral load (using quantitative real-time reverse transcription PCR) on the day before LT and 30 days after LT, respectively. Blood samples on the day before LT, representing naïve liver situations, and 30 days after LT, representing the liver graft, were stabilized following LT [19]. Changes in anti-HCV antibody titers before and after LT were recorded.

### 2.4. Detection of Anti-HCV Antibodies

Serum anti-HCV antibodies were identified using a COBAS TaqMan HCV Assay, version 2.0 (Roche Diagnostics Corporation, Indianapolis, IN, USA), with a detection limit of 25 IU/mL.

### 2.5. RT-PCR for HCV-RNA Detection

Serum RNA was extracted automatically using a COBAS AmpliPrep/COBAS TaqMan HCV Test followed by fluorescent probes for RT-qPCR amplification. The detection of HCV RNA was performed using a one-step quantitative reverse transcription polymerase chain reaction (qPCR) with a TopScript One-Step qRT-PCR Probe Kit, with a HCV qPCR probe assay and a human glyceraldehyde phosphate dehydrogenase (GAPDH) qPCR probe assay (Topgen Biotech., Kaohsiung, Taiwan), on a ViiA7 Real-Time PCR System (Applied Biosystems, Waltham, MA, USA), following the manufacturer’s instructions.

### 2.6. Immunosuppression Protocol

All recipients received the same immunosuppression protocol, being administered with mycophenolate mofetil at 250 mg Q12H PO and tacrolimus at 1 mg QD PO, and the doses were adjusted according to mycophenolic acid plasma levels > 3.0 μg/mL and tacrolimus blood concentrations of 5–10 ng/mL Post-LT anti-HCV therapy for recurrent HCV viremia was provided in accordance with practice guidelines [20]. We did not have an induction therapy other than for ABO-incompatible liver transplantation, even because of HCC-associated liver transplantation.

### 2.7. Follow-Up Parameters

Serum tests regarding hepatobiliary function were routinely monitored following LT. When abnormal laboratory tests were repeatedly measured, further investigations and imaging studies, such as a Doppler ultrasound of the liver and magnetic resonance cholangiopancreatography, were arranged depending on the persistence and severity of liver test abnormalities. In some cases, a post-LT percutaneous liver biopsy for a histological confirmation of immune-mediated damage was also performed on the basis of the clinical decision of LT surgeons [21]. ERC with biliary stenting or surgical revision is necessary and should be performed immediately when BC is identified and is the preferred treatment option. Correction in early technical revision is more important than the modulation of immunity at this moment.

### 2.8. Pathological Interpretation

The criteria for a histopathological diagnosis of allograft rejection were defined on the basis of the 1995 Banff classification, and severity grades were characterized according to the rejection activity index [22]. BCs in our study were determined clinically as a requirement of post-LT endoscopic retrograde cholangiopancreatography for biliary stenting or a surgical revision of biliary tract anastomosis and episodes of post-LT biliary tract infection requiring systemic parenteral antibiotics and hospitalization.

### 2.9. Ethics

All procedures involving human participants were performed in accordance with the ethical standards of the institutional and/or national research committee and the 1964 Helsinki Declaration and its later amendments or comparable ethical standards. The study protocol was approved and authorized by the Ethics Committee of our hospital (approval number: 202300159B0). Informed consent was obtained from all patients included in the study. We followed the Strengthening the Reporting of Observational Studies in Epidemiology statement guidelines for reporting observational studies. No allograft donors or recipients were from a vulnerable population.

### 2.10. Statistics

Statistical analyses were performed using the SPSS (version 22.0; SPSS Inc., Chicago, IL, USA) and SAS software (version 9.4; SAS Institute, Inc., Cary, NC, USA). Descriptive values are expressed as mean ± standard deviation and percentages. Categorical variables were compared using the chi-squared or Fisher’s exact test, and continuous variables were compared using Student’s *t*-test. All tests were two-tailed, and a *p*-value of <0.05 was considered statistically significant.

## 3. Results

### 3.1. Patient Characteristics

The clinical characteristics were determined for the 153 allograft recipients who were seropositive for anti-HCV antibodies. Among these recipients, 31 (20.3%) constituted the DAA group and 122 (79.7%) constituted the DAA-naïve group. The mean age at LT was 54.5 years, and men accounted for 47.7% of the population. The mean post-LT follow-up period was 43.6 months. Before LT, only one (3.2%) patient in the DAA group and 69 patients (56.6%) in the DAA-naïve group still had detectable serum HCV RNA. Those with a detectable viral load in the DAA-naïve group were patients without anti-viral therapy (n = 49, 71%) or without an effective response to interferon therapy (n = 20, 29%). Following LT, none of the patients in the DAA group had HCV viremia; 61 (50%) patients with detectable serum HCV viral loads in the DAA-naïve group required further post-LT anti-viral therapy. The majority of liver donors were living donors (136/153, 88.9%). Moreover, 73 (47.7%) allograft recipients were diagnosed as having hepatocellular carcinoma (HCC) at the time of LT, and six (4.9%) patients in the DAA-naïve group had de novo HCC following LT (Table 1).

### 3.2. Incidence of Allograft Injury: BCs and ACR

**Figure 2:**
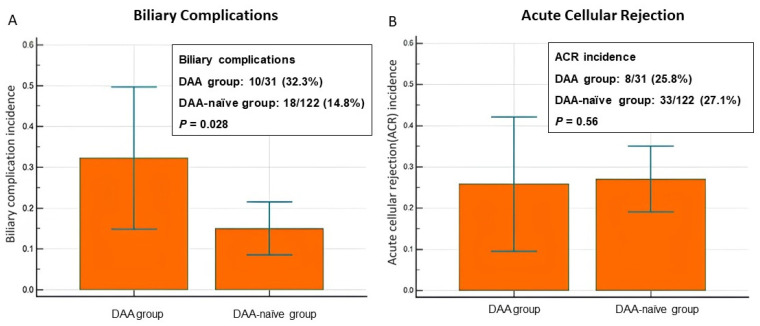
Comparison of biliary complications (**A**) and acute cellular rejection incidences (**B**) between the DAA group and DAA-naïve group.

Among the 31 patients in the DAA group, 32.3% (10/31) had BCs and 25.8% (8/31) had ACR. Of the patients in the DAA-naïve group (n = 122), 14.8% (18/122) had BCs and 27.1% (33/122) had ACR (Table 2). The incidence of BCs was higher in the DAA group than in the DAA-naïve group (32.3% vs. 14.8%, *p* = 0.028). The incidence of ACR was comparable between the two groups (25.8% vs. 27.1%, *p* = 0.56; Figure 2). Table 3 demonstrates that there were no significant differences in terms of age, sex, and graft ischemic time for recipients with or without BCs and ACR. A regression analysis was performed; however, the results are less significant. ACR appears to have the greatest explanatory power regarding the recipient age (*p* = 0.021, odds ratio = 1.09), and DAAs have little association with gender. For biliary complications, pre-LT DAAs exhibit explanatory power (*p* = 0.02, odds ratio = 2.9222).

### 3.3. Fluctuation in Anti-HCV Antibody Titers and Allograft Injury

**Figure 3:**
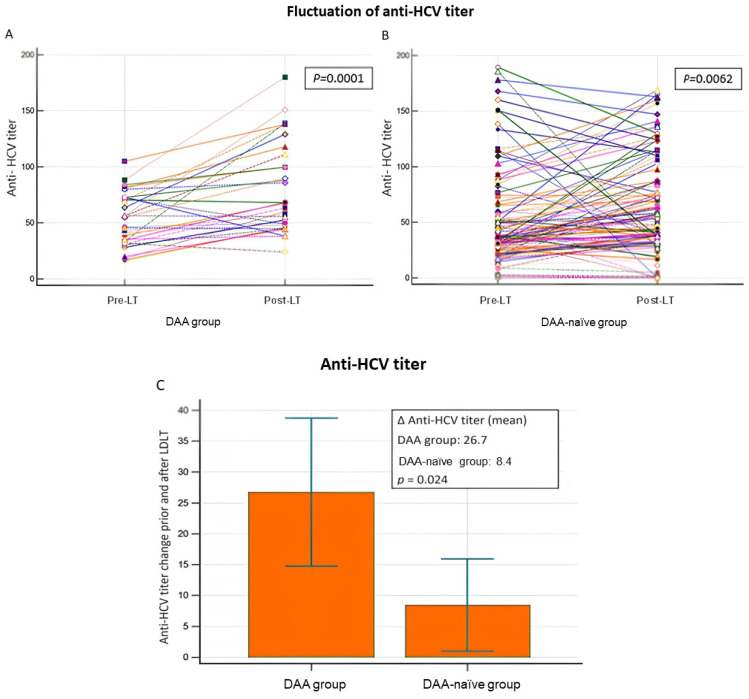
Fluctuation in anti-HCV titers (**A**,**B**) and a comparison of Δanti-HCV titers (**C**) before and after liver transplantation between the DAA group and DAA-naïve group.

In most of the 153 patients with HCV infection who received liver allografts, their post-LT anti-HCV antibody titers were upregulated (Table 4 and Figure 3A,B). Compared with the DAA-naïve group, the mean level of anti-HCV antibody titer upregulation was significantly higher in the DAA group (*p* = 0.0024; Figure 3C). Among the allograft recipients with BCs (n = 28) and ACR (n = 41), those in the DAA group had higher mean levels of anti-HCV antibody titer upregulation (*p* = 0.05 and *p* < 0.005, respectively; Table 5).

### 3.4. Multivariate Logistical Analysis of the Impact of Confounding Factors

Due to the disparity in up/downregulation, dividing BC and ACR groups will make the sample size of a single group insufficient for multivariate analysis. However, if only the ∆anti-HCV Ab titer value is directly analyzed, it is not statistically significant (Table 6). Moreover, Table 4 and Table 5, corresponding to two separate diagrams, attempt to present different information. Table 4 presents the results of the delta anti-HCV antibody titers in terms of the significant difference between the DAA and DAA-naïve groups (*p* < 0.05). Table 5 presents a comparison of the relationship between BCs and ACR in the pre-treated DAA+/− groups and up/downregulation. In addition, the so-called delta anti-HCV antibody titer divides the regulation system into up/downregulation by >0 and <0. However, the ∆anti-HCV Ab titer level itself is not significantly related to BCs and ACR. It is very difficult to present a *p*-value if you artificially divide up/downregulation, and it is meaningless to directly compare the *p*-value of up/downregulation (Table 6).

## 4. Discussion

In this study, we determined the role of anti-HCV antibodies, which are considered a useful diagnostic and screening tool for HCV infection, in an LT setting. Studies in the literature have rarely investigated antibody responses in HCV-infected recipients receiving liver allografts. According to our review of the literature, this is the first study to assess the association between changes in anti-HCV antibody titers before and after LT and allograft injury, including BCs (*p* = 0.05) and ACR (*p* < 0.005) (Table 5 and Table 6), in patients with chronic HCV who received pre-LT DAA therapy. The significant findings of this study are as follows: First, the mean level of anti-HCV antibody titer upregulation was significantly higher in the DAA group, and there was no strong correlation with post-LT HCV viremia. Second, the incidence of BCs was higher in the DAA group than in the DAA-naïve group. Furthermore, we observed that, among allograft recipients with BCs and ACR, those in the DAA group had higher mean levels of anti-HCV antibody titer upregulation following LT. The comparable incidence of ACR between the DAA and DAA-naïve groups may be explained by the difficulty in differentiating between ACR and recurrent HCV following LT [23]. In our current study, 50% (61/122) of recipients showed positive serum HCV-RNA after LT. Differentiating between ACR and early recurrent hepatitis C after LT is a challenging histological and clinical problem in the management of patients receiving transplantation for HCV-related cirrhosis. In particular, both pathological conditions are associated with lymphocytic infiltration and variable degrees of bile duct injury in the portal tracts, as well as the presence of centrilobular necrosis. Clinically, increased aminotransferase and bilirubin levels characterize both diseases, whereas serum HCV-RNA is of little help; moreover, both diseases may coexist. T-cell-depleting therapy as an induction may play a crucial role and should be investigated. In our recent study, 85% of allograft recipients with positive hepatic HCV-RNA indicate that the incidence of ACR in the DAA-naïve group was overestimated [19]. This means that part of ACR may be confounded with the pathological picture of HCV recurrence in the DAA-naïve group. Whether DAA treatment results in immune reconstitution remains unclear. Cytotoxic CD8^+^ T-cells are important mediators of immune responses regarding the disruption of immune functions via chronic HCV infection. Agatha V. et al. reported that generalized CD8^+^ T-cell dysfunction may be related to the degree of liver damage and the severity of liver fibrosis in chronic HCV infection. Furthermore, increased CD8^+^ T-cell activity does not resolve after the completion of DAA therapy and HCV clearance. This may be one of the factors involved in immune reconstitution [24]. Immune response has a complex role in the recurrence of HCC following LT in patients with hepatitis C. After LT, our recent hepatic micro-RNA study revealed that, due to factors associated with cytochrome P450, which regulates immunosuppressive drugs in the liver, fluctuations in anti-HCV antibody levels may be the result of significant changes in CD4/CD8 T-cell activity in the immune system, which is essential for preventing organ rejection or attack on cholangiocytes. These changes will affect the body’s ability to recognize and destroy biliary complications or hepatocyte mutation into cancer cells following LT, which may result in de novo or recurrent HCC. Anti-HCV antibodies can be considered detective antibodies that cannot protect a new liver graft, and fluctuations in antibody concentrations may hide the presence of the virus, thus potentially modulating the immune response and constructing a new microenvironment that supports the development or recurrence of HCC following LT. In DAA-naïve patients, the risk of developing HCC after treatment with DAAs is relatively low. Studies have shown that DAA treatment for HCV infection is associated with a reduced risk of HCC development in both cirrhotic and non-cirrhotic patients. However, the exact number of DAA-naïve patients who have developed HCC may vary across different research studies and patient populations.

The crucial role of antibody responses in HCV infection has been noted. HCV-specific T-cell dysfunction and viral escape mutations are the main factors leading to chronic HCV infection [16,25]. HCV clearance is associated with both innate immunity and adaptive immunity, including the association with CD8^+^ T-cells, CD4^+^ T-cells, and B-cells [26]; notably, antibody responses and the development of neutralizing antibodies to antiviral immunity are closely related to CD4^+^ T-cell and humoral immune responses [11,27]. Recently, numerous studies have demonstrated the reversal of HCV-specific immune response after successful viral elimination via DAA treatment [17,28]. However, changes in antibody responses, especially fluctuations in anti-HCV antibody titers before and after DAA-mediated immune responses and HCV elimination, have never been investigated. The results of the current study reveal that the mean level of post-LT anti-HCV antibody titer upregulation was significantly higher in the DAA group, suggesting the restoration of immunity to HCV infection via pre-LT DAA therapy. This clinical finding still requires further validation to establish the exact immunological mechanisms underlying anti-HCV antibody titer fluctuation and DAA therapy.

We considered chronic HCV infection in liver transplantation to be a unique model of human immunology. On the one hand, the phenomenon of the significant upregulation of anti-HCV antibody titers in the DAA group following LT might indicate immune reactivation in recipients of liver allografts; on the other hand, our study revealed that among the recipients with BCs and ACR, those in the DAA group had significantly higher mean levels of post-LT anti-HCV antibody titer upregulation. We can reasonably speculate that the reactivated immune response induced via DAA treatment, and the possible presence of high levels of IgG and immune complexes, resulted in immune-mediated BCs and ACR. BCs are among the most complex post-LT problems and often result from biliary stricture. According to previous studies, in addition to ischemia-related factors, immune-mediated injury targeting the biliary epithelium plays a major role in the development of post-LT BCs [29,30,31,32]. ACR, defined as a histological feature of portal infiltrative inflammatory cells, nonsuppurative cholangitis, and endotheliitis (Snover’s triad), is a prominent post-LT immunopathologic injury mediated by CD4^+^ T-cells [22]. To explain our clinical findings, we postulate that immunological response alteration and immune function restoration following DAA therapy might not only prompt allograft rejection, but also interfere with the healing and fibrosis processes of biliary anastomosis, consequently increasing the rate of post-LT BCs.

This study has several strengths. Although the mechanisms underlying post-DAA anti-HCV antibody titer upregulation and BCs and ACR have yet to be fully elucidated, our study adds to the body of knowledge on the possible DAA-mediated immunopathologic responses in the field of post-LT alloimmunity, and also demonstrates the significance of antibody responses to HCV infection before and after an LT. They may be involved in genetic manipulation in chronic C hepatitis infection. The genetic background plays a role in the immune response to environmental factors, such as viral infections. This response is often associated with MHC, HLA class II, and DR3/4. The proliferation of T-cells associated with immune checkpoints, whether expressed via a blockade or agonism, is closely related to the positive and negative co-stimulation of T-cell activation. Furthermore, positive co-stimulation promotes T-cell proliferation and cytokine production, prevents anergy, and facilitates the differentiation of T helper cells and cytotoxic T lymphocytes. In contrast, negative co-stimulation inhibits T-cell proliferation and cytokine production, and enhances anergy and the induction of T reg. This mechanism needs to be activated through the Fas ligand (FASL) and LIGHT pathway involving IFN gamma and IL-2 from cytotoxic T lymphocytes, as well as primary adhesion with decoy receptor 3 (DcR3), to attack the target organs. Therefore, it is evident that FASL is associated with HCV infection via DcR3, which interacts with infected hepatocytes, thus resulting in the failure of spontaneous HCV clearance. Finally, B-cells express immunoglobin to conclude the immune reaction with a so-called detective antibody (anti-HCV). This antibody not only fails to protect host hepatocytes, but also, the presence of DcR3 may cause fluctuations in the anti-HCV titer, thereby influencing the regulation of the immune response in the infected hepatocytes [33]. In particular, we discovered the relationship between HCV clearance mediated via DAA therapy, fluctuations in anti-HCV antibody titers induced via cellular and humoral immune responses, and allograft injury mediated via immune responses in the field of liver transplantation, all of which provide insights for future human research aimed at developing HCV-antibody-based vaccines to protect new liver grafts from HCV reinfection.

Our study has some limitations. First, the sample size of this study is relatively small, and the study is single-centered. Nevertheless, we consider the risk of bias to be very small due to the standardization of pre-LT clinical and laboratory assessments, sophisticated LT surgical techniques, and post-LT protocols for immunosuppression regimens and graft outcome surveillance. Second, we could determine the association between changes in anti-HCV antibody titers before and after LT procedures and allograft injury only through a retrospective review of medical records. There was a lack of data regarding the immunosuppressive treatment schedule and the serum levels of immunosuppressive drugs to enable comparisons between anti-HCV antibody titers. Future prospective studies may be warranted to both identify the role of molecular expression levels in allograft rejection and clarify the mechanisms underlying the association between DAA-induced anti-HCV antibody titer fluctuations and immune reactions to post-LT allograft injury; the findings of such studies may shed light on measures for optimizing pre-LT plans and donor selection, as well as serving as a reference for modifying anti-HCV treatment protocols for potential liver allograft recipients in order to reduce the incidence and severity of both BCs and ACR.

In conclusion, our study identified the phenomenon of the significant upregulation of anti-HCV antibody titers following liver transplantation in recipients receiving pre-LT DAA therapy. The fluctuations in anti-HCV antibody titers might have been engendered via the restoration of immunity induced through pre-LT DAA use for treating chronic HCV infection, and this immune response might have an impact on allograft injury, including BCs and ACR.

## Figures and Tables

**Table 1:** Patient profile of 153 chronic hepatitis C recipients who underwent liver transplantation.

Variable, n (%)/Median ± SD	All Patients n = 153	DAA Group n = 31 (20.3)	DAA-Naïve Group n = 122 (79.7)	*p*-Value
Sex, n (%) Male/female	72 (47.7)/81 (52.3)	12 (38.7)/19 (61.3)	60 (49.6)/62 (50.4)	0.28
Age at transplant (years), mean ± SD	54.5	57.4 ± 7.5	57.2 ± 6.6	0.85
Follow-up (months), mean ± SD	43.6	33.2 ± 18.6	46.1 ± 21.7	0.0029
Serum HCV RNA positive, n (%)				
Pre-transplant	70 (45.8)	1 (3.2)	69 (56.6)	<0.000001
Post-transplant	61 (39.9)	0 (0)	61 (50)	<0.000001
HCV genotype, n (%) 1/2/3/6/undetected	54 (35.3)/39 (25.5)/2 (1.3)/2 (1.3)/56 (36.6)	8 (25.8)/10 (32.3)/1 (3.2)/0 (0)/12 (38.7)	46 (37.7)/29 (23.8)/1 (0.8)/2 (1.6)/44 (36.1)	0.19
AFP (ng/mL), mean ± SD				
Pre-transplant	14.1 ± 70.0	11.9 ± 21.4	14.7 ± 37.8	0.69
Post-transplant	3.8 ± 7.3	4.2 ± 5.1	3.7 ± 3.2	0.52
Liver donor, n (%) Living donor/deceased donor	136 (88.9)/17 (11.1)	27 (87.1)/4 (12.9)	109 (87.3)/13 (10.7)	0.75
HCC diagnosed at LT, n (%) Absent/present	80 (52.3)/73 (47.7)	19 (61.3)/12 (38.7)	61 (50)/61 (50)	0.057
MELD score, mean ± SD	18.0 ± 18.7	17.6 ± 9.6	18.2 ± 9.4	0.75
Liver explant pathology				
Viable tumor identified, n (%)	60 (39.2)	18 (58.1)	42 (34.4)	0.11
Largest tumor (cm), mean ± SD	2.9 ± 3.0	2.6 ± 1.1	2.9 ± 1.6	0.39
Number of lesions, mean	2.5	2.3 ± 1.4	2.6 ± 1.8	0.52
Lymphovascular invasion, n (%)	16 (10.5)	3 (15.0)	13 (22.8)	0.47
Post-LT complications, n (%)				
Biliary complication	28 (18.3)	10 (32.3)	18 (14.8)	0.028
Acute cellular rejection	41 (26.8)	8 (25.8)	33 (27)	0.56
Post-LT de novo HCC/recurrence, n (%)	6 (3.9)/0	0 (0)/0 (0)	6 (4.9)/0 (0)	0.35

Student’s *t*-test (tails = 1, type = 2); chi-squared and Fisher’s exact test; *p*-value of <0.05 was considered statistically significant.

**Table 2:** Biliary complications (BCs) and acute cellular rejection (ACR) in 153 chronic C hepatitis recipients who underwent liver transplantation with/without pre-transplant DAA therapy.

Category	Anti-HCV (+) n = 153 (%)		*p*-Value
	DAA Group n = 31 (20.3)	DAA-Naïve Group n = 122 (79.7)	
Positive BC	10 (32.3)	18 (14.8)	<0.05
Negative BC	21 (67.7)	104 (85.2)	
Positive ACR	8 (25.8)	33 (27.1)	>0.05
Negative ACR	23 (74.2)	89 (73.0)	

Chi-squared test; *p*-value of <0.05 was considered statistically significant.

**Table 3:** Comparison of characteristics among recipients with BCs and ACR.

Variable/Median ± SD	BCs (+) (n = 28)	BCs (−) (n = 125)	*p*-Value	ACR (+) (n = 41)	ACR (−) (n = 112)	*p*-Value
Age (years)	58.0 ± 5.9	57.0 ± 6.9	0.49	59.6 ± 5.3	56.3 ± 7.0	0.0065
Sex (M/F)	14/14	59/66	0.84	20/21	53/59	1.00
Pre-LT DAA use (Yes/no)	10/18	21/104	0.036	8/33	23/89	1.00
Graft warm ischemic time (min)	35.4 ± 4.9	37.9 ± 6.7	0.16	36.3 ± 4.6	37.8 ± 7.0	0.33
Graft cold ischemic time (min)	71.1 ± 96.9	51.9 ± 64.6	0.33	34.3 ± 10.5	62.8 ± 81.4	0.09
Post-LT day 30						
AST (U/L)	67.9 ± 78.2	63.5 ± 211.5	0.91	53.6 ± 40.8	68.2 ± 224.5	0.69
ALT (U/L)	84.5 ± 96.0	53.6 ± 98.6	0.13	70.7 ± 58.3	55.2 ± 109.4	0.40
Total bilirubin (mg/dL)	0.92 ± 0.75	1.53 ± 7.14	0.65	0.97 ± 1.3	1.58 ± 7.5	0.61
Albumin (g/dL)	4.13 ± 0.47	4.11 ± 0.48	0.84	4.0 ± 0.59	4.1 ± 0.42	0.21
INR	1.00 ± 0.16	1.08 ± 0.34	0.30	1.03 ± 0.2	1.07 ± 0.35	0.50

Student’s *t*-test (tails = 1, type = 2); chi-squared and Fisher’s exact test; *p*-value of <0.05 was considered statistically significant.

**Table 4:** The fluctuation in pre/post-LT serum anti-HCV Ab titers in patients (n = 153) receiving liver transplantation with/without pre-transplant DAA therapy.

Category	DAA Group n = 31 (%)		DAA-Naïve Group n = 122 (%)	
**Anti-HCV Ab titer**	28.05 ± 33.96 ^a^		10.50 ± 39.93 ^a’^	
	Upregulation n = 25 (80.7)	Downregulation n = 6 (19.3)	Upregulation n = 85 (69.7)	Downregulation n = 37 (30.3)
	37.58 ± 30.18 ^b^	−11.66 ± 14.02 ^c^	27.97 ± 25.45 ^b’^	−29.62 ± 38.37 ^c’^

^a^ vs. ^a’^: *p* = 0.01301; ^b^ vs. ^b’^: *p* > 0.05; ^c^ vs. ^c’^: *p* > 0.05; Student’s *t*-test (tails = 1, type = 2); *p*-value of <0.05 was considered statistically significant.

**Table 5:** Fluctuation in serum anti-HCV Abs associated with post-LT biliary complications and acute cellular rejection before and after liver transplantation.

Category	BCs n = 28 (%)				ACR n = 41 (%)			
**Anti-HCV Ab titer** **(S/CO ratio)**	Upregulation n = 19 (67.9)		Downregulation n = 9 (32.1)		Upregulation n = 29 (70.7)		Downregulation n = 12 (29.3)	
	25.13 ± 25.39 ^a^		−20.39 ± 27.08 ^a’^		18.71 ± 18.81 ^b^		−23.18 ± 23.55 ^b’^	
	DAA (+) n = 9 (47.4)	DAA (−) n = 10 (52.6)	DAA (+) n = 1 (11.1)	DAA (−) n = 8 (88.9)	DAA (+) n = 7 (24.1)	DAA (−) n = 22 (75.9)	DAA (+) n = 1 (8.3)	DAA (−) n = 11 (91.7)
	34.87 ± 24.41 ^c^	16.36 ± 24.08 ^c’^	−2.55	−22.63 ± 28.05	34.07 ± 27.44 ^d^	13.82 ± 11.34 ^d’^	−23.14	−23.18 ± 24.71

^a^ vs. ^a’^: *p* < 0.001; ^b^ vs. ^b’^: *p* < 0.001; ^a^ vs. ^b^: *p* > 0.05; ^a’^ vs. ^b’^: *p* > 0.05; ^c^ vs. ^c’^: *p* = 0.05; ^d^ vs. ^d’^: *p* < 0.005; Student *t*-test (tails = 1, type = 2); *p*-value of <0.05 was considered statistically significant.

**Table 6:** Multivariate logistic regression analysis of the incidence difference in allograft injury between anti-HCV antibody titer up- and downregulation.

Category n (%)	DAA Group 31 (20.2)	DAA-Naïve Group 122 (79.7)	*p*-Value
∆Anti-HCV Ab titer following LT, mean ± SD	28.05 ± 33.96	10.50 ± 39.93	0.013
Post-LT positive fluctuation in anti-HCV titer, mean ± SD	37.58 ± 30.18	27.97 ± 25.45	0.714
Post-LT negative fluctuation in anti-HCV titer, mean ± SD	−11.66 ± 14.02	−29.62 ± 38.37	0.43
Post-LT positive fluctuation in anti-HCV titer, n (%)	6 (19.3)	37 (30.3)	0.27
Post-LT negative fluctuation in anti-HCV titer, n (%)	25 (80.7)	85 (69.7)	

∆Anti-HCV Ab: delta anti-HCV antibody titer divides the regulation system into up/downregulation by >0 and <0.

## Data Availability

The datasets used and analyzed in this study are available from the corresponding author upon reasonable request.
